# Antibiotic Resistance of Staphylococci from Bulk-Tank Milk of Sheep Flocks: Prevalence, Patterns, Association with Biofilm Formation, Effects on Milk Quality, and Risk Factors

**DOI:** 10.3390/biology10101016

**Published:** 2021-10-09

**Authors:** Daphne T. Lianou, Efthymia Petinaki, Peter J. Cripps, Dimitris A. Gougoulis, Charalambia K. Michael, Katerina Tsilipounidaki, Anargyros Skoulakis, Angeliki I. Katsafadou, Natalia G. C. Vasileiou, Themis Giannoulis, Chrysoula Voidarou, Vasia S. Mavrogianni, Mariangela Caroprese, George C. Fthenakis

**Affiliations:** 1 Veterinary Faculty, University of Thessaly, 43100 Karditsa, Greece; dlianou@vet.uth.gr (D.T.L.); peterjohncripps@gmail.com (P.J.C.); dgoug@vet.uth.gr (D.A.G.); cmichail@vet.uth.gr (C.K.M.); vmavrog@vet.uth.gr (V.S.M.); 2 University Hospital of Larissa, 41110 Larissa, Greece; petinaki@med.uth.gr (E.P.); tsilipoukat@gmail.com (K.T.); skulakis@gmail.com (A.S.); 3 Faculty of Public and One Health, University of Thessaly, 43100 Karditsa, Greece; agkatsaf@vet.uth.gr; 4 Faculty of Animal Science, University of Thessaly, 41110 Larissa, Greece; vasileiounat@gmail.com (N.G.C.V.); themisgia@gmail.com (T.G.); 5 Department of Agriculture, University of Ioannina, 47132 Arta, Greece; xvoidarou@uoi.gr; 6 Department of Agriculture, Food, Natural Resources and Engineering (DAFNE), University of Foggia, 71122 Foggia, Italy; mariangela.caroprese@unifg.it

**Keywords:** bulk-tank milk, mastitis, methicillin, milk, sheep, somatic cell counts, staphylococcus, tetracycline, total bacterial counts

## Abstract

**Simple Summary:**

This work investigated the presence of staphylococci resistant to antibiotics in the raw milk produced in sheep farms, which is intended for human consumption, in an extensive study performed throughout Greece. In 31% of flocks, staphylococci resistant to at least one antibiotic were recovered; in 12% of flocks, staphylococci resistant to at least three different antibiotic classes were found. Factors potentially associated with recovery of resistant isolates were the lack of experience by farmers (<5 years), the period immediately post lambing, and the intensive management system applied in the flocks.

**Abstract:**

The objectives of this work were to study prevalence and characteristics of resistance to antibiotics of staphylococcal isolates from the bulk-tank milk of sheep flocks across Greece, to assess possible associations of the presence of antibiotic resistance with the quality of milk in these flocks and to evaluate flock-related factors potentially associated with antibiotic resistance among these isolates. A cross-sectional study was performed in 325 sheep flocks in Greece. Bulk-tank milk samples were collected for bacteriological examination; staphylococcal isolates were evaluated for resistance to 20 antibiotics. Oxacillin-resistant staphylococcal isolates, isolates resistant to any antibiotic, and multi-resistant isolates were recovered from 8.0%, 30.5%, and 12.0% of flocks, respectively. Of 232 isolates, 11.6% were resistant to oxacillin, 46.1% were resistant to at least one antibiotic, and 16.4% were multi-resistant. Resistance was seen more frequently among coagulase-negative (50.6%) than among *Staphylococcus aureus* (31.5%) isolates. Resistance was more frequent against penicillin and ampicillin (34.1% of isolates), clindamycin (17.7%), and fosfomycin (14.2%). An association was found between biofilm formation by staphylococci and resistance to fosfomycin. For recovery of oxacillin-resistant isolates, the lack of experience by farmers emerged as a significant factor; respective factors for the isolation of staphylococci resistant to any antibiotic or multi-resistant isolates were the early stage of the lactation period (0th–1st month) and the intensive management system applied in the flocks, respectively.

## 1. Introduction

Sheep farming for milk production is an important sector of the Greek agricultural industry. The country has a significant annual sheep milk production, which amounted to 639,165,000 L in 2019 [1]. This quantity accounts for around 20% of European and 7% of world sheep milk production [2]. Sheep milk is used for cheese production. Various cheese types are produced from sheep milk, and an important proportion (e.g., ‘feta’ cheese) is exported, thus highlighting the international significance of sheep milk production in Greece.

The quality of raw milk is important, as it contributes to the quality of cheese produced, which has a significance for public health. Among the various factors that account for milk quality of raw milk are antibiotic-resistant bacteria.

Staphylococci are the most frequently recovered bacteria from the milk of sheep flocks [3,4]. Previous studies that examined staphylococcal isolates from bulk-tank milk of sheep farms mostly studied resistance to methicillin, with contrasting results: for example, in Italy, Macori et al. [5] reported that 53% of 51 *S. aureus* recovered from the milk of six sheep farms showed resistance to methicillin, whilst Giacinti et al. [6] reported that the prevalence of methicillin-resistant *S. aureus* among 286 sheep farms was only 0.7%. Furthermore, in Greece, Papadopoulos et al. [7] indicated that 99.5% of *S. aureus* isolates from 36 sheep farms in only one part of the country were resistant to methicillin. In studies in which more antibiotics were evaluated, the proportion of resistant isolates was found to be between 48.5% and 83% in Spain [8,9] and 70% in Jordan [10]. So far, relevant studies have focused on the characteristics of the staphylococcal isolates and have not dealt with the identification of within-flock variables that may be associated with the development of resistance.

The objectives of this work were (a) to study the prevalence and the patterns of resistance to antibiotics of staphylococcal isolates from bulk-tank milk of sheep flocks across Greece, (b) to assess possible associations of the presence of antibiotic resistance with the quality of milk in these flocks, and (c) to evaluate flock-related factors potentially associated with the presence of antibiotic resistance among these staphylococcal isolates.

## 2. Materials and Methods

### 2.1. Sheep Flocks and Sampling

**Figure 1:**
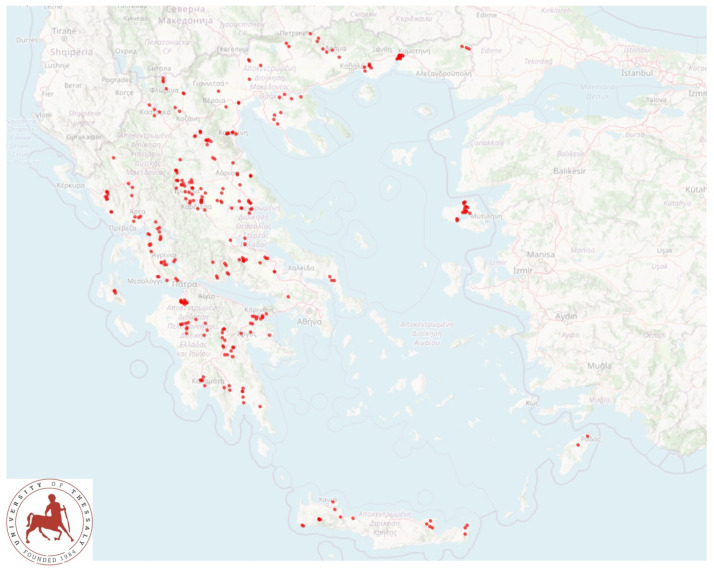
Location of 325 sheep flocks around Greece, which were visited for bulk-tank milk sampling.

A cross-sectional study involving 325 flocks was performed from April 2019 to July 2020 and covered all the 13 administrative regions of Greece (Figure 1). Flocks were included into the study on a convenience basis (willingness of shepherds to accept a visit by university personnel for interview and sample collection), as detailed previously [4]. The principal investigators (authors D.T.L. and G.C.F.) accompanied by other investigators visited all the flocks for sample collection.

Initially, the management practices applied in the flocks were recorded during an interview with the shepherd by means of a detailed questionnaire [11]. Bulk-tank milk samples were taken aseptically from each flock for somatic cell counting, milk composition evaluation, and bacteriological examinations. The samples were collected directly from the milk cooling tank in each farm; all tanks had one or more agitators which stirred the milk continuously until the cover of the tank was raised for sample collection. Milk samples were collected by sterile plastic single-use pipettes which were immersed into the tank to withdraw the samples. A total of four samples were collected from the milk tank of each flock, and a new pipette was used for each sample. Immediately after collection, samples were transferred into sterile plastic Universal-type vials. Samples were packed at 0.0 to 4.0 °C and transported for laboratory examinations [4].

### 2.2. Laboratory Examinations

Of the four milk samples collected from the milk tank of each flock, two samples were used for somatic cell counts (SCC) and milk composition measurement; the other two were used for the bacteriological examinations. Two subsamples were created and processed from each of the four samples, such that each separate test was performed four times (each one in different subsamples).

SCC and milk composition measurement were performed within 4 h of collection, whilst bacteriological examinations started within 24 h of sample collection [4]. Bacteriological examinations from each of the four relevant subsamples included total bacterial counts (TBC), performed by employing the standardized procedures described by Laird et al. [12], and culturing on *Staphylococcus*-selective medium (Mannitol salt agar; BioPrepare Microbiology, Athens, Greece) for aerobic incubation at 37 °C for 48 h; if there was no growth, media were re-incubated for a further 24 h. After completion of sample aliquot withdrawal for microbiological examination, the temperature of the respective samples was measured and was found to never exceed 3.8 °C.

Bacterial isolation and initial identification by means of Gram staining and evaluation of catalase production were performed using standard methods [13,14]. Definite identification of the staphylococcal isolates to a species level was performed using matrix-assisted laser desorption/ionization time-of-flight mass spectrometry (VITEK MS; BioMerieux, Marcy-l’-Étoile, France).

Then, in vitro biofilm formation by the staphylococcal isolates was evaluated. This was performed by using a combination of (a) the culture appearance on Congo Red agar plates and (b) the results of a microplate adhesion test. The procedures were detailed by Vasileiou et al. [15] for staphylococcal isolates recovered from sheep milk.

Lastly, the susceptibility testing to 20 antibiotics (amikacin, ampicillin, ceftaroline, ciprofloxacin, clindamycin, erythromycin, fosfomycin, fucidic acid, gentamicin, linezolid, moxifloxacin, mupirocin, mupirocin high level, oxacillin, penicillin G, rifampin, teicoplanin, tetracycline, tobramycin, and trimethoprim–sulfamethoxazole) was performed by means of the automated system BD Phoenix™ M50 (BD Diagnostic Systems, Sparks, MD, USA). The interpretation of the results was based on criteria of the European Committee on Antimicrobial Susceptibility Testing (EUCAST) (http://www.eucast.org).

### 2.3. Data Management and Analysis

#### 2.3.1. Data Management

Presence of staphylococci in the bulk-tank milk was defined by the isolation of ≥3 colonies of the same staphylococcal species on at least one agar plate of the four that were cultured with a subsample from each bulk-tank milk from a flock.

Biofilm formation by the staphylococcal isolates was indicated by the combination of the results of the two methods (culture appearance on Congo Red agar and microplate adhesion) (Table S1) [15], and staphylococcal strains were then characterized as biofilm-forming or non-biofilm-forming.

Based on the results of susceptibility/resistance testing, isolates were classified as susceptible, susceptible to increased exposure, or resistant to each antibiotic according to the EUCAST criteria. As no ‘susceptible to increased exposure’ isolates were found, this possible result was omitted from the analyses. Multidrug-resistant isolates were those found resistant to at least three different classes of antibiotics [16].

During cell counting, total bacterial counting, and milk composition measurement, for each bulk-tank milk sample, the results of the two subsamples from each sample were averaged, and then the two means were again averaged for the final result regarding each bulk-tank milk.

SCCs were transformed to somatic cell scores (SCS) [17,18] by using the following formula: SCS = log_2_(SCC/100) + 3, and TBCs were transformed to log_10_; for both parameters, the transformed data were used in the analyses. The transformations were conducted in order to normalize the raw SCC and use a measure that adjusts and weights samples appropriately. For the presentation of results, the transformed findings were back-transformed as follows: 100 × 2^(SCS−3)^ for SCC and 10^log^ for TBC data.

#### 2.3.2. Statistical Analysis

Data were entered into Microsoft Excel and analyzed using SPSS v. 21 (IBM Analytics, Armonk, NY, USA). Basic descriptive analysis was performed. Exact binomial confidence intervals (CI) were obtained.

Twenty-five variables were evaluated for potential association with recovery of staphylococcal isolates resistant to antibiotic from the bulk-tank milk of these flocks (Appendix A); these were either taken directly from the answers of the interview performed at the start of the visit or calculated on the basis of these answers. For each of these variables, categories were created according to the answers of the farmers.

The outcomes of “isolation of oxacillin-resistant staphylococcal isolates from the bulk-tank milk” and “isolation of resistant staphylococcal isolates from the bulk-tank milk” (i.e., isolates resistant to any (at least one) antibiotic) were considered. Exact binomial CIs were obtained. Initially, the importance of predictors was assessed by using cross-tabulation with Pearson’s chi-square test and with simple logistic regression. Subsequently, multivariable models were created, initially offering to the model all variables, which achieved a significance of *p* < 0.2 in the univariable analysis. Variables were removed from the initial model by backward elimination. The *p*-value of removal of a variable was assessed by the likelihood ratio test, and, for those with a *p*-value >0.2, the variable with the largest probability was removed. This process was repeated until no variable could be removed with a *p*-value >0.2. The variables required for the final multivariable models are shown in Table S2.

Subsequently, the outcome of “isolation of multi-resistant staphylococcal isolates from the bulk-tank milk” was considered. Only the variables that achieved *p* < 0.2 in the analysis for isolation of resistant staphylococci, were evaluated and the same procedures as above (i.e., univariable and multivariable analyses) were performed. The variables required for the final multivariable model are shown in Table S2.

Lastly, the potential association of isolation of a resistant staphylococcal isolate with SCC, TBC, and composition of bulk-tank milk was assessed by using one-way analysis of variance.

In all analyses, statistical significance was defined at *p* ≤ 0.05.

## 3. Results

### 3.1. Staphylococcal Recovery and Presence of Antibiotic Resistance

Staphylococci were recovered from bulk-tank milk samples from 206 flocks (63.4%, 95% CI: 58.0–68.4%) (*Staphylococcus aureus* and coagulase-negative staphylococci were isolated from samples from 54 (16.6%) and 164 (50.5%) flocks, respectively). A total of 232 staphylococcal isolates were obtained (54 *S. aureus* and 178 coagulase-negative staphylococci) from the samples (Table 1).

Resistant (to any (at least one) antibiotic) or multi-resistant staphylococcal isolates were recovered from 99 (30.5%, 95% CI: 25.7–35.7%) or 40 (12.3%, 95% CI: 9.2–16.3%) flocks, respectively.

Of the 232 staphylococcal isolates, 107 (46.1%, 95% CI: 39.8–52.6%) (17 *S. aureus* and 90 coagulase-negative isolates, *p* = 0.014 for comparison between *S. aureus* and coagulase-negative staphylococci, *p* = 0.0003 for comparison between the various coagulase-negative species) were found to be resistant to antibiotics. Furthermore, 41 isolates (17.7%, 95% CI: 13.3–23.1%) (two *S. aureus* and 39 coagulase-negative isolates, *p* = 0.002 for comparison between *S. aureus* and coagulase-negative staphylococci, *p* < 0.0001 for comparison between the various coagulase-negative species) were found to be multi-resistant. Details are in Table 1.

At the isolate level, resistance was recorded more frequently against penicillin and ampicillin (79 isolates, 34.1% of all isolates), clindamycin (41 isolates, 17.7% of all isolates), fosfomycin (33, 14.2%), tetracycline (28, 12.1%), oxacillin (27, 11.6%), and erythromycin (21, 9.1%) (Table S3).

At flock level, isolates resistant to penicillin and ampicillin were recovered from 75 (23.1%, 95% CI: 18.8–28.0%) flocks, to clindamycin from 40 (12.3%, 95% CI: 9.2–16.3%) flocks, to fosfomycin from 33 (10.2%, 95% CI: 7.3–13.9%) flocks, to tetracycline or oxacillin from 26 (8.0%, 95% CI: 5.5–11.5%) flocks, and to erythromycin from 21 (6.5%, 95% CI: 4.3–9.7%) flocks.

Among the staphylococcal species, *S. aureus* was found to be resistant more frequently to ampicillin, penicillin, and tetracycline (7/54, 7/54, and 6/54 isolates, respectively), *S. equorum* was found to be resistant more frequently to ampicillin, penicillin, and erythromycin (15/23, 15/23, and 14/23 isolates, respectively), *S. haemolyticus* was found to be resistant more frequently to ampicillin, penicillin, and tetracycline (8/22, 8/22, and 3/22, respectively), and *S. lentus* was found to be resistant more frequently to ampicillin, clindamycin, oxacillin, and penicillin (11/12 for the four agents) (Table S3).

### 3.2. Biofilm Formation

Of the 232 isolates, 165 (71.1%, 95% CI: 65.0–76.6%) were found to be biofilm-forming. Overall, no association was seen between biofilm formation and resistance to antibiotics. Of the 107 resistant isolates, 77 (72.0%, 95% CI: 62.8–79.6%) (13 *S. aureus* and 64 coagulase-negative isolates) were biofilm-forming (*p* = 0.79). Furthermore, of the 41 multi-resistant isolates, 33 (80.5%, 95% CI: 66.0–89.8%) (two *S. aureus* and 31 coagulase-negative isolates) were biofilm-forming (*p* = 0.14).

An association was found specifically for resistance to fosfomycin; 30/33 isolates resistant to fosfomycin (90.9%) were found to be biofilm-forming (*p* = 0.007). With regard to the other antibiotics against which resistance was frequently recorded (penicillin and ampicillin, clindamycin, tetracycline, oxacillin, and erythromycin), no such association was evident (*p* > 0.14 for all comparisons) (Table S4).

A tendency for association was found for *S. kloosii*; 5/5 resistant isolates were found to be biofilm-forming (*p* = 0.09). For the other species, no association was seen (*p* > 0.16 for all comparisons).

### 3.3. Associations with Milk Quality

The mean fat content of the bulk-tank milk from which resistant staphylococcal isolates were recovered was lower than that of bulk-tank milk from which no resistant staphylococci were isolated: 6.03% ± 0.08% vs. 6.22% ± 0.06% (mean ± standard error of the mean) (*p* = 0.05). Moreover, there was a tendency for total bacterial counts in the bulk-tank milk from which multi-resistant staphylococcal isolates were recovered to be higher than those of bulk-tank milk from which no resistant staphylococci were isolated: 639 × 10^3^ (95% CI: 380 × 10^3^–1096 × 10^3^) cfu mL^−1^) vs. 373 × 10^3^ (95% CI: 309 × 10^3^–447 × 10^3^) cfu·mL^−1^ (*p* = 0.07). No other association of milk quality with the isolation of resistant or multi-resistant staphylococci was evident (Table S5).

### 3.4. Variables Associated with Isolation of Resistant Staphylococcal Isolates from the Bulk-Tank Milk

#### 3.4.1. Isolation of Oxacillin-Resistant Staphylococcal Isolates

During the univariable analysis, a significant association with isolation of oxacillin-resistant staphylococcal isolates from the bulk-tank milk was evident for only one of the 25 variables evaluated (Table S6). This was the length of previous animal farming experience of the farmer.

**Figure 2:**
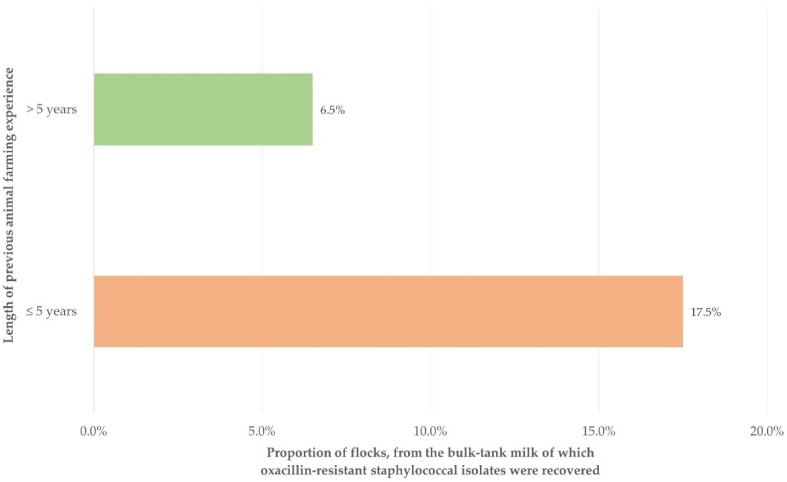
Proportion of flocks from which oxacillin-resistant staphylococcal isolates were recovered, in accordance with the length of previous animal farming experience of farmers in these flocks.

Among the variables included in the multivariable analysis (Tables S2 and S6), the following emerged to be the only significant factor: length of previous animal farming experience of the farmer (*p* = 0.018) (Figure 2 and Table 2).

#### 3.4.2. Isolation of Staphylococcal Isolates Resistant to at Least One Antibiotic

During the univariable analysis, a significant association with isolation of resistant staphylococcal isolates from the bulk-tank milk was evident for four of the 25 variables evaluated (Table S7). These were the following: management system applied in the flock, month into the lactation period at sampling, means of calculating live bodyweight for the administration of pharmaceutical products, and presence of working staff in the flock.

**Figure 3:**
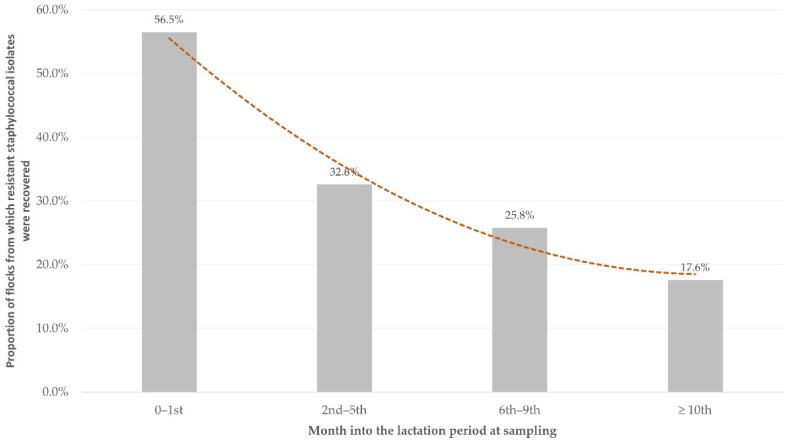
Proportion of flocks from which resistant staphylococcal isolates were recovered from the bulk-tank milk, in accordance with the stage of the lactation period of animals in these flocks (dashed line: trendline).

Among the variables included in the multivariable analysis (Tables S2 and S7), only the following emerged to be the only significant factor: month into the lactation period at sampling (*p* = 0.003) (Figure 3 and Table 3).

#### 3.4.3. Isolation of Multi-Resistant Staphylococcal Isolates

During the univariable analysis, a significant association with isolation of multi-resistant staphylococcal isolates from the bulk-tank milk was evident for four of the 10 variables evaluated (Table S8). These were the following: management system applied in the flock, age of lamb removal from their dam, use of teat disinfection after milking, and length of previous animal farming experience of the farmer.

**Figure 4:**
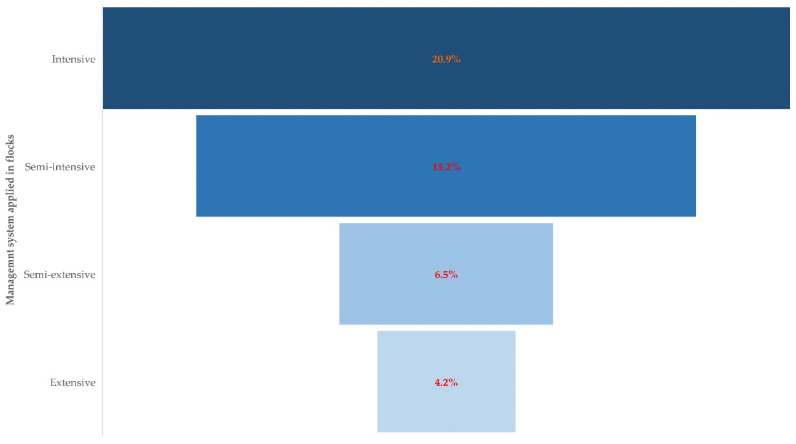
Proportion of flocks from which multi-resistant staphylococcal isolates were recovered, in accordance with the management system applied in these flocks (dashed line: trendline).

Among the variables included in the multivariable analysis (Tables S2 and S8), the following emerged to be the only significant factor: management system applied in the flock (*p* = 0.015) (Figure 4 and Table 4).

## 4. Discussion

A scientific opinion of the European Food Safety Authority [19] pointed out the importance of antibiotic resistance of bacteria isolated from raw milk for the public health. Hence, there is a scope to study the patterns of such resistance in sheep farms. Moreover, the evaluation and identification of predictors related to the management in the farms would help to implement procedures in the farms, which may contribute to limiting the presence of antibiotic resistance.

This study included sheep flocks from all parts of Greece. In this way, conditions prevailing throughout the country were taken into account, and factors of regional importance weighed less.

### 4.1. Presence of Antibiotic Resistance in Staphylococcal Isolates

With regard to the variety and the frequency of staphylococcal species recovered, it is noteworthy that we performed an extensive field study to identify causal agents of staphylococcal mastitis in sheep in Greece [3,20]. That study, which was in the same area as the current work (thus making the results comparable to a large extent), found that *S. simulans*, *S. chromogenes*, and *S. epidermidis* predominated, whilst *S. equorum*, *S. capitis*, and *S. lentus* were recovered much less frequently. This suggests that many of the isolates recovered from the raw milk in the current study may have originated from sources outside the animals. Apart from the mammary gland of the ewes, i.e., as agents of intramammary infection, these bacteria could have originated from the udder and teat skin and the surface of equipment for milk handling and storage (including teatcups, pipelines of the milking parlor, and milk tank) [21]. Moreover, in flocks in which hand-milking is applied, they might have also originated from the hands of the milkers [22].

The extent of antibiotic resistance was in general at the same level as that reported in other relevant reports from the para-Mediterranean region, where dairy sheep are kept and milk is produced for human consumption. The results of the present study showed low-level resistance among *S. aureus* isolates, but a significantly greater problem among the coagulase-negative isolates. Indeed, the prevalence of oxacillin-resistant *S. aureus* isolates in the sheep flocks (0.6%) is lower than the global prevalence rate of MRSA contamination of raw milk from small ruminants, which in a large international meta-analysis study was reported to be 1.1% [23]. *S. aureus* is a significant causal agent of clinical mastitis in sheep, which can be diagnosed easily and can be followed by the instigation of effective treatment. In contrast, coagulase-negative isolates cause disease of lesser severity (e.g., subclinical mastitis, which is difficult to diagnose and only infrequently treated), in addition to being present in the environment or part of a bacterial carrier state in animals [24]; as a consequence, there are more opportunities for exposure to factors leading to the development of resistance. These results are in line with those of a recent study that we performed on the antibiotic resistance patterns of ovine mastitis pathogens, in which *S. aureus* also showed significantly less frequent resistance than the coagulase-negative isolates [25]. It is also possible that some of the coagulase-negative isolates might have originated from humans (e.g., farm personnel), given that some species (e.g., *S. hominis* or *S. haemolyticus*) are confirmed human pathogens. Moreover, the detection of resistance to fosfomycin, which is not licensed for veterinary use, further supports that some of the recovered isolates likely were of human origin.

### 4.2. Association of Antibiotic Resistance with Biofilm Formation

Biofilm formation by bacteria is considered a significant mechanism that can lead to bacterial survival during antibiotic administration and failure of treatment. In general, biofilm formation is considered to promote dissemination of antibiotic resistance. In *S. aureus*, biofilm formation has been found to increase the transfer of plasmid-borne determinants of resistance [26] and is associated with the presence of more antibiotic resistance genes [27]. Furthermore, staphylococci present in biofilm communities show higher evolutionary rates, due to the oxidative stress prevailing therein; this contributes to the development of resistance through spontaneous mutations followed by the vertical dissemination of resistance genes [28].

The present results confirmed the above for fosfomycin, for which an association of resistance with biofilm formation was seen. Fosfomycin has a bactericidal action, belonging to the class of phosphonic antibiotics. It acts by inhibition of biogenesis of the bacterial cell wall, specifically by inactivating the enzyme UDP-*N*-acetylglucosamine-3-enolpyruvyltransferase. It is a phosphoenolpyruvate analogue that inhibits the above enzyme by alkylating an active site cysteine residue, after entering the bacterial cell through the glycerophosphate transporter [29]. The antibiotic has a broad spectrum of in vitro activity against Gram-positive bacteria, including methicillin-resistant *S. aureus* and vancomycin-resistant *Enterococcus*, and Gram-negative organisms, including *Pseudomonas aeruginosa*, extended-spectrum *β*-lactamase (ESBL) pathogens, and carbapenem-resistant Enterobacteriaceae. Although fosfomycin is an older antibiotic (it was discovered in 1969 and received approval for use by the Food and Drug Administration of the United States of America in 1996), it is a safe drug that can be useful in the presence of increased prevalence of multi-resistant pathogens.

A possible mechanism for our findings involves the *glpT* gene, which encodes for the glycerol-3-phosphate/fosfomycin symporter [30,31]. Under in vitro conditions, deletion of *glpT* considerably increased biofilm formation by the mutant strains [32]; moreover, increased antibacterial activity and efficacy of fosfomycin were attributed to elevated expression of GlpT, which led to increased uptake of the drug and its subsequent intracellular accumulation [33], whilst deletion of *glpT* in *S. aureus* led to an increase in fosfomycin’s minimum inhibitory concentrations, which promotes antibiotic resistance [34]. Thus, we postulate that the negative regulation of fosfomycin’s uptake, mediated mainly by *glpT* expression, can lead to resistant phenotypes and increased biofilm formation.

### 4.3. Possible Effects on Milk Quality

Some differences in milk quality were found to be associated with the recovery of resistant staphylococcal isolates from the milk. These can occur because of difficulties in treating intramammary infections in the flocks, where resistance has developed. For example, intramammary infection in ewes can result in reduced fat content in their milk [35], which can be reflected in a lower fat content in the bulk-tank milk; moreover, the increased total bacterial counts may also reflect a difficulty in treating cases of mastitis, due to the presence of resistant isolates or possibly the development of resistance by relevant bacteria in the farm.

### 4.4. Predictors for Antibiotic-Resistant Staphylococcal Isolates

The significance of the (short) length of farming experience in the recovery of resistant isolates reflects the lack of receiving information from the extensive campaigns carried out in Greece throughout the last 30 years, aiming to inform people regarding the importance of preventing development of resistance to antibiotics. Greece has one of the highest frequencies of resistance, found alike in community and hospital strains [36], and many public or private organizations have set up relevant campaigns within their areas of responsibility. One such campaign was staged by the Hellenic Veterinary Association [37], with leaflets produced for farmers to inform them about the importance of resistant bacterial isolates for the animals, as well as about the potential transmission to humans. Moreover, discussions of veterinarians with farmers would include highlighting the importance of preventing antibiotic resistance. It is, therefore, likely that farmers with shorter experience would not be fully aware of the importance of the problem and might be using practices and procedures that promote the development of resistance.

The major factor for the presence of resistance to at least one antibiotic was found to be the month of the lactation period (with increased recovery of resistant isolates at the early stages). Increased use of antibiotics is often made in the early stages of the lactation period, and this could have contributed significantly. Major reasons for this are (i) the presence of growing lambs in the farm, which may be affected by infections requiring antibiotic treatment (e.g., respiratory infections [38]), and (ii) an increased incidence of mastitis that requires the frequent administration of antibiotics for its treatment [39]. During early lactation, sheep also spend more time indoors, sometimes with high animal densities within the barns, and this favours the spread of bacteria, including resistant isolates, which may later contaminate the bulk-tank milk. Lastly, during the first 2 months postpartum, there is increased incidence of mastitis, consequently to the transition from lamb sucking to milking [20,40], which leads to increased antibiotic administration to ewes in that period.

An ‘intensive management system’ can include various aspects of flock husbandry and organization (e.g., machine-milking, housing facilities, and nutrition), which can contribute to the development of mastitis in ewes [41,42] and, thus, an increased use of antibiotics for treatment and consequent resistance development by causal organisms. Frequent use of disinfectants can also be applied in intensively managed flocks, and this can be associated with the development of antibiotic resistance [43,44]), because animals are housed and there is a need for the repeated cleaning of animal houses and equipment.

## 5. Conclusions

The presence of resistant staphylococcal isolates in the milk produced for human consumption on sheep farms raises issues within the ‘one health’ concept. Recent studies have shown that cell-free genetic material of staphylococci resistant to antibiotics, not destroyed during thermal processing of milk, could be transferred to humans [45,46]. Such resistance genes could be incorporated in other bacteria, part of the normal flora of humans, thus leading to the dissemination of resistance genes. This process could provide a way for the resistance genes of staphylococci present in the raw milk to be disseminated through the dairy products. This puts further pressure on limiting the staphylococcal presence in milk and preventing the development of resistance in dairy sheep farms, with a view to minimizing public health concerns.

The current findings focused on exploring management factors in sheep farms, which can be associated with the presence of resistant isolates in the raw milk produced therein. The findings can be employed as a guide to limit development of resistance by applying good practices that contribute to preventing development of such resistance within the ‘one health’ concept.

## Figures and Tables

**Table 1:** Frequency of staphylococcal species recovered from bulk-tank milk of 325 sheep flocks in Greece.

Staphylococcal Species	Frequency of Staphylococcal Isolates		
	All Isolates ^1^	Resistant Isolates ^2,3^	Multi-Resistant Isolates ^3,4^
*Staphylococcus aureus*	54 (0.233)	17 (0.315)	2 (0.037)
*Staphylococcus simulans*	35 (0.151)	4 (0.114)	0 (0.000)
*Staphylococcus equorum*	23 (0.099)	17 (0.739)	14 (0.609)
*Staphylcoccus haemolyticus*	22 (0.095)	11 (0.500)	1 (0.045)
*Staphylococcus chromogenes*	13 (0.056)	4 (0.308)	1 (0.077)
*Staphylococcus lentus*	12 (0.051)	11 (0.917)	8 (0.667)
*Staphylococcus lugdunensis*	11 (0.047)	7 (0.636)	1 (0.091)
*Staphylococcus warneri*	9 (0.039)	3 (0.333)	1 (0.111)
*Staphylococcus kloosii*	7 (0.030)	5 (0.714)	4 (0.571)
*Staphylococcus capitis*	6 (0.026)	3 (0.500	0 (0.000)
*Staphylococcus intermedius*	6 (0.026)	3 (0.500)	0 (0.000)
*Staphylococcus cohnii* subsp. *cohnii*	4 (0.017)	3 (0.750)	1 (0.250)
*Staphylococcus epidermidis*	4 (0.017)	4 (1.000)	0 (0.000)
*Staphylococcus saprophyticus*	4 (0.017)	2 (0.500)	2 (0.500)
*Staphylococcus xylosus*	4 (0.017)	3 (0.750)	3 (0.750)
*Staphylococcus auricularis*	3 (0.013)	2 (0.667)	0 (0.000)
*Staphylococcus cohnii* subsp. *urealyticum*	3 (0.013)	2 (0.667)	1 (0.333)
*Staphylococcus sciuri*	3 (0.013)	1 (0.333)	1 (0.333)
*Staphylococcus vitulinus*	3 (0.013)	1 (0.333)	1 (0.333)
*Staphylococcus hominis*	2 (0.009)	1 (0.500)	0 (0.000)
*Staphylococcus pasteuri*	2 (0.009)	2 (1.000)	0 (0.000)
*Staphylococcus carnosus*	2 (0.009)	1 (0.500)	0 (0.000)
Total	232 (1.000)	107 (0.461)	41 (0.177)

^1^ In brackets: proportion of isolates of the species among all isolates; ^2^ resistant to any (at least one) antibiotic; ^3^ in brackets: proportion of resistant or multi-resistant isolates among the isolates of the respective species; ^4^ multi-resistant isolates: those found resistant to at least three different classes of antibiotics [16].

**Table 2:** Results of multivariable analysis for isolation of oxacillin-resistant staphylococcal isolates from the bulk-tank milk of 325 sheep flocks in Greece.

Variable (*n* = 1)	Odds Ratio ^1^ (95% Confidence Intervals)	*p*
Length of previous animal farming experience of farmers		0.018
≤5 years (*n* = 74)	2.747 (1.202–6.276)	0.017
>5 years (*n* = 251)	Reference	

^1^ Odds ratio calculated against the lowest prevalence associations of the variables.

**Table 3:** Results of multivariable analysis for isolation of resistant staphylococcal isolates from the bulk-tank milk of 325 sheep flocks in Greece.

Variable (*n* = 1)	Odds Ratios ^1^ (95% Confidence Intervals)	*p*
Month into the lactation period at sampling		0.003
0th to 1st (*n* = 23)	6.067 (1.361–27.050)	0.018
2nd to 5th (*n* = 138)	2.258 (0.617–8.259)	0.218
6th to 9th (*n* = 147)	1.627 (0.443–5.973)	0.463
Subsequently to 9th (*n* = 17)	Reference	

^1^ Odds ratios calculated against the lowest prevalence associations of the variables.

**Table 4:** Results of multivariable analysis for isolation of multi-resistant staphylococcal isolates from the bulk-tank milk of 325 sheep flocks in Greece.

Variable (*n* = 1)	Odds Ratios ^1^ (95% Confidence Intervals)	*p*
Management system applied in the flock		0.015
Intensive (*n* = 43)	13.939 (1.745–111.329)	0.013
Semi-intensive (*n* = 151)	3.512 (0.449–27.460)	0.23
Semi-extensive (*n* = 107)	1.859 (0.221–15.606)	0.57
Extensive (*n* = 24)	Reference	

^1^ Odds ratios calculated against the lowest prevalence associations of the variables.

## Data Availability

Most data presented in this study are in the Supplementary Materials. The remaining data are available on request from the corresponding author. The data are not publicly available as they form part of the PhD thesis of the first author, which has not yet been examined, approved, and uploaded in the official depository of PhD theses from Greek Universities.

## Supplementary Materials

The following are available online at www.mdpi.com/article/10.3390/biology10101016/s1: Table S1. Characterization of staphylococcal strains recovered from milk samples of ewes classified according to biofilm formation, through a combination of results of culture appearance on Congo Red agar and microplate adhesion method; Table S2. Details of multivariable models employed for the evaluation of the isolation of resistant staphylococcal isolates from the bulk-tank milk of 325 sheep flocks in Greece; Table S3. Frequency of susceptibility/resistance to individual antibiotics of staphylococcal isolates recovered from bulk-tank milk of 325 sheep flocks in Greece; Table S4. Details of associations of antibiotic resistance with biofilm formation by staphylococcal isolates from the bulk-tank milk of 325 sheep flocks in Greece; Table S5. Details of associations of milk quality with isolation of resistant or multi-resistant staphylococcal isolates from the bulk-tank milk of 325 sheep flocks in Greece; Table S6. Results of univariable analysis for association with isolation of oxacillin-resistant staphylococcal isolates from the bulk-tank milk of 325 sheep flocks in Greece; Table S7. Results of univariable analysis for association with isolation of staphylococcal isolates resistant to at least one antibiotic from the bulk-tank milk of 325 sheep flocks in Greece; Table S8. Results of univariable analysis for association with isolation of multi-resistant staphylococcal isolates from the bulk-tank milk of 325 sheep flocks in Greece.

## Appendix A

**Table A1:** Variables (*n* = 25) evaluated for potential association with presence of antibiotic resistance in staphylococci isolated from the bulk-tank milk of 325 sheep flocks in Greece.

Management system applied in the flock (description according to EFSA classification) [47]
Month into the lactation period at sampling (month)
Machine- or hand-milking (description)
No. of ewes in the flock (no.)
Total milk quantity per ewe obtained during the preceding milking period (litres)
Average number of lambs born per ewe (no.)
Collaboration with a veterinarian (yes/no)
Total visits made annually by veterinarians to the flock during the preceding season (no.)
Clinical mastitis annual incidence risk in the flock (%)
Age of lamb removal from their dams (days)
Daily number of milking sessions (no.)
Duration of the dry-period (months)
Means of calculating live bodyweight for the administration of pharmaceutical products (weighing/estimation)
Routine overdosing (compared to dose prescribed) of pharmaceuticals (yes/no)
Annual frequency of systemic disinfections in the farm (no. of occasions)
Routine administration of antimicrobials in newborns (yes/no)
Vaccination against mastitis (yes/no)
Administration of ‘dry-ewe’ treatment at the end of the lactation period (yes/no)
Use of teat disinfection after milking (yes/no)
Age of the farmer (years)
Length of previous animal farming experience of the farmer (years)
Education of the farmer (description)
Farmer by profession (yes/no)
Family tradition in farming (yes/no)
Presence of working staff in the flock (yes/no)
