# Supplementary material for: Antibiotic Resistance of Staphylococci from Bulk-Tank Milk of Sheep Flocks: Prevalence, Patterns, Association with Biofilm Formation, Effects on Milk Quality, and Risk Factors
Source: Biology (Basel). 2021 Oct 9;10(10):1016. doi: 10.3390/biology10101016 (PMC8533144; doi:10.3390/biology10101016)
Supplement: Supplementary file 1 [file biology-10-01016-s001.zip › biology-1402306-supplementary.pdf]

Antibiotic Resistance of Staphylococci from Bulk-Tank Milk of Sheep Flocks: Prevalence, Patterns, Association with Biofilm-Formation, Effects on Milk Quality, Risk Factors

Daphne T. Lianou, Efthymia Petinaki, Peter J. Cripps, Dimitris A. Gougoulis, Charalambia K. Michael, Katerina Tsilipounidaki, Anargyros Skoulakis, Angeliki I. Katsafadou, Natalia G.C. Vasileiou, Themis Giannoulis, Chrysoula Voidarou, Vasia S. Mavrogianni, Mariangela Caroprese and George C. Fthenakis

Table S1. Characterisation of staphylococcal strains recovered from milk samples of ewes classified according to biofilm-formation, by combination of results of culture appearance on Congo Red agar and microplate adhesion method.

		**Sum of results of each of three separate testing lots for each strain in the microplate adhesion method** [1]			
		**0**	**1**	**2**	**3**
**Culture appearance on Congo Red agar**	-	non BF [2]	non BF	non BF	weak BF
	±	non BF	non BF	BF	BF
	+	weak BF	BF	BF	BF

[1] sum ranged from 0 (i.e., in all three testing lots median, of three triplicates, OD was $<$ [0.85 × median OD of the positive control strain]) to 3 (i.e., in all three testing lots median, of three triplicates, OD was $\geq$ [0.85 × median OD of the positive control strain]) (OD: optical density) [Vasileiou et al 2018]; [2] biofilm-forming.

Reference

Vasileiou, N.G.C.; Chatzopoulos, D.C.; Gougoulis, D.A.; Sarrou, S.; Katsafadou, A.I.; Spyrou, V.; Mavrogianni, V.S.; Petinaki, E.; Fthenakis, G.C. Slime-producing staphylococci as causal agents of subclinical mastitis in sheep. *Vet. Microbiol.* **2018**, *224*, 93–99

Table S2. Details of multivariable models employed for the evaluation of the isolation of resistant staphylococcal isolates from the bulk-tank milk of 325 sheep flocks in Greece.

Outcome	Variables offered to the multivariable models (*n*)	Variables required in the final models
Isolation of an oxacillin-resistant staphylococcal isolate from the bulk-tank milk	7	(a) routine administration of antimicrobials in newborns, (b) vaccination against mastitis, (c) length of previous animal farming experience of the farmer
Isolation of a resistant staphylococcal isolate from the bulk-tank milk	10	(a) management system applied in the flock, (b) month into the lactation period at sampling, (c) annual frequency of systemic disinfections in the farm, (d) length of previous animal farming experience of the farmer, (e) presence of working staff in the flock
Isolation of a multi-resistant staphylococcal isolate from the bulk-tank milk	7	(a) management system applied in the flock, (b) length of previous animal farming experience of the farmer

Table S3. Frequency of susceptibility / resistance to individual antibiotics [1] of staphylococcal isolates recovered from bulk-tank milk of 325 sheep flocks in Greece

	n	AMP	AZI	CXI	CIP	CLA	CLI	ERY	FOS	FUS	GEN	MOX	MUP	OXA	PEN	RIF	TEI	TET	TOB	SXT
S. aureus	54	7	0	0	1	0	2	1	0	1	2	0	1	2	7	0	0	6	1	2
S. simulans	35	1	0	0	0	0	0	0	3	0	0	0	0	1	1	0	0	0	0	0
S. equorum	23	15	0	0	0	0	11	14	12	1	0	0	0	1	15	0	0	2	0	0
S. haemolyticus	22	8	0	0	0	0	1	0	2	0	0	0	0	0	8	0	0	3	1	0
S. chromogenes	13	3	0	0	0	0	1	1	0	0	0	0	0	0	3	0	0	3	0	0
S. lentus	12	11	0	0	1	0	11	3	1	2	0	1	0	11	11	0	0	1	0	0
S. lugdunensis	11	6	0	0	0	0	1	0	1	0	0	0	0	0	6	0	0	2	0	0
S. warneri	9	3	0	0	0	0	1	0	2	0	0	0	0	0	3	0	0	1	0	0
S. kloosii	7	5	0	0	0	0	4	0	4	0	0	0	0	4	5	0	0	1	0	0
S. capitis	6	2	0	0	0	0	0	0	3	0	0	0	0	0	2	0	0	1	0	0
S. intermedius	6	2	0	0	0	0	0	0	0	0	0	0	0	1	2	0	0	1	0	0
S. cohnii ssp. *cohnii*	4	2	0	0	0	0	2	1	0	2	0	0	0	1	2	0	0	1	0	0
S. epidermidis	4	3	0	0	0	0	0	1	0	0	0	0	0	1	3	0	0	1	0	0
S. saprophyticus	4	2	0	0	0	0	0	0	2	2	0	0	0	0	2	0	0	2	0	0
S. xylosus	4	2	0	0	0	0	3	0	0	3	0	0	0	1	2	1	0	0	0	0
S. auricularis	3	0	0	0	0	0	0	0	2	0	0	0	0	0	0	0	0	1	0	0
S. cohnii ssp. *urealyticum*	3	2	0	0	0	0	1	0	0	1	0	0	0	1	2	0	0	0	0	0
S. sciuri	3	1	0	0	0	0	1	0	0	1	0	0	0	1	1	0	0	0	0	0
S. vitulinus	3	1	0	0	0	0	1	0	0	1	0	0	0	1	1	0	0	1	0	0
S. hominis	2	0	0	0	0	0	1	0	0	0	0	0	0	0	0	0	0	0	0	0
S. pasteuri	2	2	0	0	0	0	0	0	1	0	0	0	0	0	2	0	0	1	0	0
S. carnosus	2	1	0	0	0	0	0	0	0	0	0	0	0	1	1	0	0	0	0	0

Total	232	79	0	0	2	0	41	21	31	14	2	1	1	27	79	1	0	28	2	2

[1] as established by use of VITEK 2; [2] AMP: ampicillin, AZI: azithromycin, CXI: cefoxitin, CIP: ciprofloxacin, CLA: clarithromycin, CLI: clindamycin, ERY: erythromycin, FOS: fosfomycin, FUS: fucidic acid, GEN: gentamicin, MOX: moxifloxacin, MUP: mupirocin, OXA: oxaxillin, PEN: penicillin, RIF: rifampicin, TEI: teicoplanin, TET: tetracycline, TOB: tobramycin, SXT: trimethoprim-sulfamethoxazole.

Table S4. Details of associations of antibiotic resistance with biofilm-formation by staphylococcal isolates from the bulk-tank milk of 325 sheep flocks in Greece.

Antibiotic	Proportion of biofilm-forming resistant isolates	*p*
All antibiotics	77/107 (72.0%)	0.79
Ampicillin, penicillin	56/79 (70.9%)	0.95
Clindamycin	33/41 (80.5%)	0.14
Fosfomycin	30/33 (90.9%)	0.007
Tetracycline	21/28 (75.0%)	0.63
Oxacillin	21/27 (77.8%)	0.42
Erythromycin	16/21 (76.2%)	0.59

Table S5. Details of associations of milk quality with isolation of resistant or multi-resistant staphylococcal isolates from the bulk-tank milk of 325 sheep flocks in Greece.

**Isolation of resistant staphylococcal isolates from the bulk-tank milk (*n* = 99)**	**No isolation of resistant staphylococcal isolates from the bulk-tank milk (*n* = 226)**	***p***
Somatic cell counts (cells mL^{-1})		
0.527×10^6 (95% CI: 0.461×10^6 - 0.600×10^6)	0.473×10^6 (95% CI: 0.430×10^6 - 0.518×10^6)	0.21
Flocks with increased somatic cell counts (> 1.000×10^6 cells mL^{-1})		
22/99 (22.2%)	32/226 (14.2%)	0.07
Total bacterial counts (cfu mL^{-1})		
499×10^3 (95% CI: 363×10^3 - 692×10^3)	360×10^3 (95% CI: 288×10^3 - 447×10^3)	0.10
Flocks with increased total bacterial counts (> 1500×10^3 cfu mL^{-1})		
18/99 (18.2%)	40/226 (17.7%)	0.92
Fat (%)		
6.03 ± 0.08	6.22 ± 0.06	0.05
Protein (%)		
4.46 ± 0.03	4.41 ± 0.02	0.10
**Isolation of multi-resistant staphylococcal isolates from the bulk-tank milk (*n* = 39)**	**No isolation of multi-resistant staphylococcal isolates from the bulk-tank milk (*n* = 286)**	***p***
Somatic cell counts (cells mL^{-1})		
0.502×10^6 (95% CI: 0.393×10^6 - 0.638×10^6)	0.487×10^6 (95% CI: 0.448×10^6 - 0.529×10^6)	0.93
Flocks with increased somatic cell counts (> 1.000×10^6 cells mL^{-1})		
8/40 (20.0%)	46/285 (16.1%)	0.54
Total bacterial counts (cfu mL^{-1})		
639×10^3 (95% CI: 380×10^3 - 1096×10^3)	373×10^3 (95% CI: 309×10^3 - 447×10^3)	0.07
Flocks with increased total bacterial counts (> 1500×10^3 cfu mL^{-1})		
7/40 (17.5%)	51/285 (17.9%)	0.95
Fat (%)		
5.99 ± 0.14	6.19 ± 0.05	0.16
Protein (%)		
4.48 ± 0.05	4.42 ± 0.01	0.17

Table S6. Results of univariable analysis for association with isolation of oxacillin-resistant staphylococcal isolates from the bulk-tank milk of 325 sheep flocks in Greece.

Isolation of oxacillin-resistant staphylococcal isolates from the bulk-tank milk (*n* = 26)				No isolation of resistant staphylococcal isolates from the bulk-tank milk (*n* = 299)				*p*
**Management system applied in the flock**								
Intensive	Semi-intensive	Semi-extensive	Extensive	Intensive	Semi-intensive	Semi-extensive	Extensive	
3	17	6	0	40	134	101	24	0.17
**Month into the lactation period at sampling**								
0–1st	2nd–5th	6th–9th	After 9th	0–1st	2nd–5th	6th–9th	After 9th	
4	12	9	1	19	126	138	16	0.30
**Machine- or hand-milking**								
Machine-milking	Hand-milking			Machine-milking	Hand-milking			
23	3			232	67			0.19
**No. of ewes in the flock**								
≤ 165 ewes	166-330 ewes	331-500 ewes	> 500 ewes	≤ 165 ewes	166-330 ewes	331-500 ewes	> 500 ewes	
4	11	8	3	84	109	58	48	0.48
**Total milk quantity per ewe obtained during the preceding milking period**								
≤ 200 L	201-400 L	> 400 L		≤ 200 L	201-400 L	> 400 L		
11	14	1		163	126	10		0.49
**Average number of lambs born per ewe**								
≤ 1.50	> 1.50			≤ 1.50	> 1.50			
22	4			257	42			0.85
**Collaboration with a veterinarian**								
Yes	No			Yes	No			
23	3			254	45			0.63
**Total visits made annually by veterinarians to the flock during the preceding season**								
≤ 4	5 - 7	> 7		≤ 4	5 - 7	> 7		
9	7	10		130	79	90		0.61
**Clinical mastitis annual incidence risk in the flock**								
≤ 0.50%	> 0.50%			≤ 0.50%	> 0.50%			
2	24			54	245			0.18
**Age of lamb removal from their dams**								
< 45 days	45–60 days	> 60 days		< 45 days	45–60 days	> 60 days		
12	12	2		107	158	34		0.55

<table>
<tr><td colspan="13">Daily number of milking sessions</td></tr>
<tr><td colspan="2">1</td><td colspan="2">2</td><td colspan="2">3</td><td colspan="2">1</td><td colspan="2">2</td><td colspan="2">3</td><td></td></tr>
<tr><td colspan="2">0</td><td colspan="2">19</td><td colspan="2">7</td><td colspan="2">1</td><td colspan="2">245</td><td colspan="2">53</td><td>0.49</td></tr>
<tr><td colspan="13">Duration of the dry-period</td></tr>
<tr><td colspan="3">≤ 2 months</td><td colspan="3">> 2 months</td><td colspan="3">≤ 2 months</td><td colspan="3">> 2 months</td><td></td></tr>
<tr><td colspan="3">10</td><td colspan="3">16</td><td colspan="3">97</td><td colspan="3">202</td><td>0.53</td></tr>
<tr><td colspan="13">Means of calculating live bodyweight for the administration of pharmaceutical products</td></tr>
<tr><td colspan="3">Weighing</td><td colspan="3">Estimation</td><td colspan="3">Weighing</td><td colspan="3">Estimation</td><td></td></tr>
<tr><td colspan="3">5</td><td colspan="3">21</td><td colspan="3">68</td><td colspan="3">231</td><td>0.68</td></tr>
<tr><td colspan="13">Routine overdosing (compared to dose prescribed) of pharmaceuticals</td></tr>
<tr><td colspan="3">Yes</td><td colspan="3">No</td><td colspan="3">Yes</td><td colspan="3">No</td><td></td></tr>
<tr><td colspan="3">7</td><td colspan="3">19</td><td colspan="3">54</td><td colspan="3">245</td><td>0.27</td></tr>
<tr><td colspan="13">Annual frequency of systemic disinfections in the farm</td></tr>
<tr><td colspan="2">0 – 1 occasion</td><td colspan="2">2 – 10 occasions</td><td colspan="2">> 10 occasions</td><td colspan="2">0 – 1 occasion</td><td colspan="2">2 – 10 occasions</td><td colspan="2">> 10 occasions</td><td></td></tr>
<tr><td colspan="2">4</td><td colspan="2">20</td><td colspan="2">2</td><td colspan="2">75</td><td colspan="2">208</td><td colspan="2">16</td><td>0.51</td></tr>
<tr><td colspan="13">Routine administration of antimicrobials in newborns</td></tr>
<tr><td colspan="3">Yes</td><td colspan="3">No</td><td colspan="3">Yes</td><td colspan="3">No</td><td></td></tr>
<tr><td colspan="3">3</td><td colspan="3">23</td><td colspan="3">67</td><td colspan="3">232</td><td>0.19</td></tr>
<tr><td colspan="13">Vaccination against mastitis</td></tr>
<tr><td colspan="3">Yes</td><td colspan="3">No</td><td colspan="3">Yes</td><td colspan="3">No</td><td></td></tr>
<tr><td colspan="3">14</td><td colspan="3">12</td><td colspan="3">112</td><td colspan="3">187</td><td>0.10</td></tr>
<tr><td colspan="13">Administration of ‘dry-ewe’ treatment at the end of the lactation period</td></tr>
<tr><td colspan="3">Yes</td><td colspan="3">No</td><td colspan="3">Yes</td><td colspan="3">No</td><td></td></tr>
<tr><td colspan="3">5</td><td colspan="3">21</td><td colspan="3">48</td><td colspan="3">251</td><td>0.67</td></tr>
<tr><td colspan="13">Use of teat disinfection after milking</td></tr>
<tr><td colspan="3">Yes</td><td colspan="3">No</td><td colspan="3">Yes</td><td colspan="3">No</td><td></td></tr>
<tr><td colspan="3">4</td><td colspan="3">22</td><td colspan="3">48</td><td colspan="3">251</td><td>0.93</td></tr>
<tr><td colspan="13">Age of the farmer</td></tr>
<tr><td colspan="3">Up to 50 years</td><td colspan="3">Over 50 years</td><td colspan="3">Up to 50 years</td><td colspan="3">Over 50 years</td><td></td></tr>
<tr><td colspan="3">16</td><td colspan="3">10</td><td colspan="3">181</td><td colspan="3">118</td><td>0.92</td></tr>
<tr><td colspan="13">Length of previous animal farming experience of the farmer</td></tr>
<tr><td colspan="3">≤ 5 years</td><td colspan="3">> 5 years</td><td colspan="3">≤ 5 years</td><td colspan="3">> 5 years</td><td></td></tr>
<tr><td colspan="3">11</td><td colspan="3">15</td><td colspan="3">63</td><td colspan="3">236</td><td>0.013</td></tr>
</table>

**Education of the farmer**				
Primary, secondary or post-secondary education	Tertiary education	Primary or secondary education	Tertiary education	
22	4	260	39	0.74
**Farmer by profession**				
Yes	No	Yes	No	
23	3	269	30	0.69
**Family tradition in farming**				
Yes	No	Yes	No	
22	4	261	38	0.69
**Presence of working staff in the flock**				
Yes	No	Yes	No	
13	13	110	189	0.18

Table S7. Results of univariable analysis for association with isolation of staphylococcal isolates resistant to at least one antibiotic from the bulk-tank milk of 325 sheep flocks in Greece.

**Isolation of resistant staphylococcal isolates from the bulk-tank milk (*n* = 99)**				**No isolation of resistant staphylococcal isolates from the bulk-tank milk (*n* = 226)**				***p***
**Management system applied in the flock**								
Intensive	Semi-intensive	Semi-extensive	Extensive	Intensive	Semi-intensive	Semi-extensive	Extensive	
17	53	26	3	26	98	81	21	0.032
**Month into the lactation period at sampling**								
0–1st	2nd–5th	6th–9th	After 9th	0–1st	2nd–5th	6th–9th	After 9th	
13	45	38	3	10	93	109	14	0.015
**Machine- or hand-milking**								
Machine-milking	Hand-milking			Machine-milking	Hand-milking			
83	16			172	54			0.12
**No. of ewes in the flock**								
≤ 165 ewes	166-330 ewes	331-500 ewes	> 500 ewes	≤ 165 ewes	166-330 ewes	331-500 ewes	> 500 ewes	
20	36	24	19	68	84	42	32	0.28
**Total milk quantity per ewe obtained during the preceding milking period**								
≤ 200 L	201-400 L	> 400 L		≤ 200 L	201-400 L	> 400 L		
49	47	3		125	93	8		0.57
**Average number of lambs born per ewe**								
≤ 1.50	> 1.50			≤ 1.50	> 1.50			
85	14			195	31			0.92
**Collaboration with a veterinarian**								
Yes	No			Yes	No			
82	17			195	31			0.42
**Total visits made annually by veterinarians to the flock during the preceding season**								
≤ 4	5 - 7	> 7		≤ 4	5 - 7	> 7		
42	20	37		97	66	63		0.13
**Clinical mastitis annual incidence risk in the flock**								
≤ 0.50%	> 0.50%			≤ 0.50%	> 0.50%			
17	82			39	187			0.73
**Age of lamb removal from their dams**								
< 45 days	45–60 days	> 60 days		< 45 days	45–60 days	> 60 days		
43	43	13		76	127	23		0.11
**Daily number of milking sessions**								
1	2	3		1	2	3		
0	81	18		1	183	42		0.80

**Duration of the dry-period**						
≤ 2 months	> 2 months		≤ 2 months	> 2 months		
33	66		74	152		0.92
**Means of calculating live bodyweight for the administration of pharmaceutical products**						
Weighing	Estimation		Weighing	Estimation		
15	84		58	168		0.037
**Routine overdosing (compared to dose prescribed) of pharmaceuticals**						
Yes	No		Yes	No		
18	81		43	183		0.86
**Annual frequency of systemic disinfections in the farm**						
0 – 1 occasion	2 – 10 occasions	> 10 occasions	0 – 1 occasion	2 – 10 occasions	> 10 occasions	
4	32	3	75	196	15	0.09
**Routine administration of antimicrobials in newborns**						
Yes	No		Yes	No		
20	79		50	176		0.70
**Vaccination against mastitis**						
Yes	No		Yes	No		
39	60		87	139		0.88
**Administration of ‘dry-ewe’ treatment at the end of the lactation period**						
Yes	No		Yes	No		
20	79		33	193		0.21
**Use of teat disinfection after milking**						
Yes	No		Yes	No		
20	79		32	194		0.17
**Age of the farmer**						
Up to 50 years	Over 50 years		Up to 50 years	Over 50 years		
61	38		136	90		0.81
**Length of previous animal farming experience of the farmer**						
≤ 5 years	> 5 years		≤ 5 years	> 5 years		
30	69		44	182		0.032
**Education of the farmer**						
Primary, secondary or post-secondary education	Tertiary education		Primary or secondary education	Tertiary education		
89	10		193	33		0.27

**Farmer by profession**				
Yes	No	Yes	No	
89	10	203	23	0.98
**Family tradition in farming**				
Yes	No	Yes	No	
84	15	199	27	0.43
**Presence of working staff in the flock**				
Yes	No	Yes	No	
48	51	75	151	0.009

Table S8. Results of univariable analysis for association with isolation of multi-resistant staphylococcal isolates from the bulk-tank milk of 325 sheep flocks in Greece.

**Isolation of multi-resistant staphylococcal isolates from the bulk-tank milk (*n* = 40)**				**No isolation of multi-resistant staphylococcal isolates from the bulk-tank milk (*n* = 286)**				***p***
**Management system applied in the flock**								
Intensive	Semi-intensive	Semi-extensive	Extensive	Intensive	Semi-intensive	Semi-extensive	Extensive	
9	23	7	1	34	128	100	23	0.030
**Month into the lactation period at sampling**								
0–1st	2nd–5th	6th–9th	After 9th	0–1st	2nd–5th	6th–9th	After 9th	
6	17	14	3	17	121	133	14	0.14
**Machine- or hand-milking**								
Machine-milking	Hand-milking			Machine-milking	Hand-milking			
35	5			220	65			0.14
**Total visits made annually by veterinarians to the flock during the preceding season**								
≤ 4	5 - 7	> 7		≤ 4	5 - 7	> 7		
19	6	15		120	80	85		0.21
**Age of lamb removal from their dams**								
< 45 days	45–60 days	> 60 days		< 45 days	45–60 days	> 60 days		
22	13	5		97	157	31		0.021
**Means of calculating live bodyweight for the administration of pharmaceutical products**								
Weighing	Estimation			Weighing	Estimation			
9	31			64	221			0.99
**Annual frequency of systemic disinfections in the farm**								
0 – 1 occasion	2 – 10 occasions	> 10 occasions		0 – 1 occasion	2 – 10 occasions	> 10 occasions		
18	74	8		61	154	10		0.12
**Use of teat disinfection after milking**								
Yes	No			Yes	No			
11	29			41	244			0.034
**Length of previous animal farming experience of the farmer**								
≤ 5 years	> 5 years			≤ 5 years	> 5 years			
15	25			59	226			0.018
**Presence of working staff in the flock**								
Yes	No			Yes	No			
18	22			105	180			0.32
